# Mapping out the Trajectory of Islamic Perspectives on Neuroethics

**DOI:** 10.1007/s41649-022-00203-8

**Published:** 2022-02-11

**Authors:** Noorina Noorfuad

**Affiliations:** College of Islamic Studies, Hamad Bin Khalifa University, Doha, Qatar

**Keywords:** Neuroethics, Neuroscience, Islam, Religion

## Abstract

The advancements of medical technology incited multi-disciplinary discussions with regard to its ethical implications. Within the neuroscientific domain, the term ‘neuroethics’ has gained prominence over recent years. However, the contributions of religious perspectives in the nascent field of neuroethics are particularly few. The scarce literature on Islamic perspectives on neuroethics merely questioned its importance and introduced a *sharia*-based framework that can be implemented. Building upon this, the possible trajectories of Islamic perspectives on neuroethics can be mapped out by tapping into several issues within Islamic bioethics, such as cloning and genomics. Topics such as these are deliberated through the collective discussions by contemporary Muslim scholars and biomedical scientists, thus producing well-informed dialogues and decisions. Building upon these may assist in developing further the Islamic perspectives on issues within neuroethics. This lays the groundwork for all the stakeholders involved in advance, in order to predict and prevent potential harms and challenges.

## Introduction

With the advancement of neuroscience in recent years, medical technology such as functional magnetic resonance imaging (fMRI) initiated a series of discussions relating to its ethical implications. These discussions do not exclusively belong within the domains of biomedical sciences but permeate across several disciplines such as philosophy and theology. Hence, there has been a need for experts to address such ever-expanding issues from their respective fields. An example of this is a particular study on the ethical aspects of neuroimaging from the Islamic perspective (Al-Delaimy 2012). Al-Delaimy expressed in his paper the need to establish an ethical framework to tackle future challenges posed by the advancements of science to predict and thus prevent potential harms. Furthermore, he emphasized on the importance of accommodating religious ethical perspectives in the field of neuroethics.

Following his paper is a commentary by Ebrahim Moosa, an Islamic studies professor at the University of Notre Dame, who echoed Al-Delaimy on the advancements of science and the efforts of Muslim scholars in providing the Islamic viewpoint on modern issues (Moosa 2012). Since neuroethics is relatively an emerging field, research pertaining to its religious and philosophical viewpoints by theologians and philosophers has not been adequately produced. By assessing questions on the relationship between religion and neuroethics, its relevance and importance, and on the ethical framework that can be applied to this field, this essay aims to map out the possible trajectories of the discussions relating to the Islamic perspectives on neuroethics.

## Islamic Bioethics and Neuroethics

As the theme of this essay focuses on the Islamic viewpoint, it is useful to refer to the overarching field of Islamic bioethics which has developed solid foundations over recent years. Among the main objectives of Islamic bioethics is to address complex issues that arise from the modern crossroads of religion and science. This proves to be a complexity that requires thorough investigations as it involves both human and divine relations (Sachedina 2009). In addition to this, newly emerged bioethical issues such as brain death and assisted reproductive technologies (ART) pose numerous challenges for contemporary Muslim scholars. This is because these issues are categorized as *nawāzil*, meaning novel cases in which clear directives relating to such topics within the primary scriptural sources (the Holy Quran and the Prophetic traditions) as well as the early and late Islamic scholarly traditions are absent (Ghaly 2015).

The religious ethical framework, *sharia*, plays a major underlying role throughout all disciplines within the Islamic tradition. It is at the forefront of Islamic bioethics as it ensures that these novel issues conform to the higher objectives of the *sharia*, namely the preservation of religion, life, lineage, intellect, and wealth. These can be observed to be in consistent with the objectives of medicine that strive for the maintenance of health, continuity of life, and preservation of the mind (Ghaly 2016). Thus, to tackle these concerns and address them within the confines of the *sharia*, scholars engage in the process of independent religio-ethical reasoning, *ijtihad*, to ascertain the deliberations and rulings on specific matters (Ghaly 2018). These will generally be produced as religious guidelines (*fatwa*) either individually by scholars or institutionally by religious councils. To ensure that these bioethical issues are contemplated carefully, religious scholars and medical scientists collaborate on the process of *ijtihad*, termed as ‘collective *ijtihad*’, to produce a well-informed discussion and decision. There are several international Muslim institutions that primarily engage in organizing conferences to facilitate collective *ijtihad* and collating *fatwas* on modern matters relating to religion and science through their publications. To name a few are the Islamic Organization for Medical Sciences (IOMS) based in Kuwait, International Islamic *Fiqh* Academy (IIFA), and Islamic *Fiqh* Academy (IFA), both based in Saudi Arabia (Ghaly 2010).

*Fatwas* on death by neurological criteria have been thoroughly debated and produced over the years. However, discussions specifically from the neuroethical standpoint and its inquiries by these institutions are currently non-existent, although it will inevitably pervade future dialogues. For neuroethics, one can anticipate the complexity of exploring its structure from the religious viewpoint as it encompasses both neuroscience of ethics and the ethics of neuroscience (Roskies 2002) and even beyond, thus extending its scope across various social structures. As personal beliefs and moral principles interact with modern science, ethical dilemmas arise from the imminent ambiguity that comes with the rapid progression of science and medical technology. Therefore, it is important to eliminate as much vagueness as possible through tackling potential challenges brought upon emerging scientific domains such as neuroethics (Greely 2012).

## Relevance and Importance

The importance of neuroethics can be observed through several national brain initiatives. The emphasis on neuroethics by the Korea Brain Initiative comes alongside its priority in advancing neuroscientific research (Jeong et al. 2019). Similar to this, in line with its developmental objectives for the neurotechnological industry, the Australian Brain Initiative regards neuroethics as vital in ensuring its success (Carter et al. 2019).

Nevertheless, discussions on neuroethics will continue to evolve as neuroscience advances. An aspect of the studies on neuroscience delves deeper into centuries-old questions on human behavior and moral responsibility. Whether man possess freewill has constantly been debated among societies across time. Thus, it is evident that the scale of impact neuroethics will ultimately have over the personal and social dimensions is extremely significant. As an example, the criminal justice system which assesses judgments of human actions and behavior could tremendously transform with the integration of neuroscience and neurotechnology in the courtrooms. For instance, concepts such as brain defense cases, neuroprediction and its relation to criminal recidivism, and neurocriminology with regard to the identification of neurobiological markers on specific behavioral traits have begun to permeate neuroscientific research over twenty years ago (Coppola 2018).

The brain defense cases studied by law and philosophy professor, Nita Farahany, are criminal cases in which neurobiological data is submitted as evidence to appeal for lighter sentences for the defendants. Farahany observed in her research the striking increase of brain defense cases that she classified as ‘a trend that’s not going away’ (Davis 2017). In contrast to this, another point under the theme of neuroethics and law are the concepts of neuroprediction and neurocriminology, which opens the possibility of determining the tendency of anti-social and violent behavior through neuroimaging (Poldrack et al. 2018). These pose significant challenges in dealing with moral conflicts involving individuals and the public at large. While the advancements of neuroscience may enhance justice systems like the accuracy of recidivism rate, it may also incur violation of rights and discrimination on individuals (Coppola 2018).

From the Islamic standpoint, such issues would undeniably pose serious complications within Islamic courts as well. Such complexities would emerge during the evaluation of cases and the determining of criminal liability in instances where modern tools are employed (Zakariyah 2017). In a section of his paper, Moosa (2012) contemplated on whether neuroethics require the viewpoint from the Islamic perspective. To this, he replied neither in the affirmative nor negative, and simply stated that ‘it depends.’ However, exploring into the recent developments of neuroethics and its challenges as reiterated above would tend to heavily result in favor of a multi-disciplinary, comprehensive, and richer neuroethical discourse. In pursuit of this, it may be useful to glance into what can be expected of the Islamic perspective on neuroethics.

## Possible Trajectories of Islamic Neuroethics

For neuroethics, its Islamic perspective can be approached through two elements: the roles of the decision-makers involved and the framework of the neuroethical discussions. As observed from the numerous processes of collective *ijtihad* by medical experts and religious scholars within the discipline of Islamic bioethics, it is evident that the role of scientists and healthcare professionals extends beyond the informative into the normative (Ghaly 2015). This is apparent in a paper by Ghaly titled ‘Collective religio-scientific discussions on Islam and HIV/AIDS’ (2013) in which he elaborated on the three-dimensional role of the biomedical scientists who participated in the symposiums on HIV/AIDS conducted by Islamic organizations such as the IIFA and the IOMS. Although the scope of both Islamic bioethics and neuroethics transcends across the scientific, religious, and philosophical domains, the roles of the decision-makers relating to neuroethical deliberations may be reversed from that of Islamic bioethics.

Pertaining to the neuroethical framework, similar to the objective of this essay is a study titled ‘A preliminary insight into an Islamic mechanism for neuroethics’ (Baharuddin et al. 2016). It introduced a *sharia*-based framework that can be implemented onto the study of neuroethics through the Islamic perspective. Maintaining the essence and importance of *sharia* as emphasized by the mentioned study, this essay differs by suggesting a deeper and detailed approach to Islamic neuroethics. By relying on specific topics that have been covered within Islamic bioethics, this section will use them as a reference in which possible trajectories of Islamic neuroethics can be mapped out. Two specific topics, namely cloning and genomics, will be briefly addressed from the Islamic bioethical perspective as well as the specific inferences relating to their potential contribution to the neuroethical discourse.

i. Cloning
  From the discussions of human cloning within Islamic bioethics, a unique and interesting point surrounds the term ‘hypothesizers’ (*al-ara’aytiyyūn*). This term which emerged in the past carried a negative connotation as people of that specific period occupied themselves with discussions on imaginary scenarios while avoiding existing problems. As human cloning has not been realized, to avoid being associated as hypothesizers and the practicality of such debates questioned, scientist Hassan Hathout expressed the need to adopt the approach of the hypothesizers in these modern times. He supported his argument by stressing on the rapid progression of science that require scholars to constantly cope with its developments (Ghaly 2010). From this, two points can be made regarding neuroethics and its Islamic perspective. Firstly, this essay and the study by Baharuddin et al. (2016) partially reflect the approach of the hypothesizers by attempting to chart out the framework of the neuroethics division within the Islamic bioethical discourse and its potential challenges. Secondly, dilemmas raised both in the domains of neuroethics and cloning posit a certain degree of complexity due to their broad extent of influence that cut across various facets of the human experience. Therefore, a deeper examination of the Islamic bioethical debates on cloning may suggest insights on the Islamic viewpoint on neuroethics.
ii. Genomics

Within the field of genetics and genomics, discussions on its ethical implications by the collective efforts of Muslim medical experts and religious scholars first took place in 1993, following the initiation of the Human Genome Project a few years earlier. Since then, more than ten symposiums on the topic were organized over the years by various institutions across the Muslim world. From these, the importance of maintaining genomic research under control and within the confines of the *sharia* was continually emphasized (Ghaly 2018), in line with the launch of the Qatar Genome Program and the Saudi Genome Program.

Several key points such as the management of incidental findings were raised with regard to the religio-ethical implications associated with genomic studies from the Islamic perspective (Ghaly et al. 2016). Along with this are the issues of privacy and the notion of freewill that also pervade the discussions of neuroethics. These points that have been thoroughly debated and developed by religious scholars, scientists, and philosophers based on the various topics within bioethics could guide and be adapted into the subsequent deliberations on the field of neuroethics.

## Conclusion

While it is apparent that this discussion pertaining to the Islamic perspective on neuroethics is still in its infancy, it emphasizes the rapid advancements of science and modern technology, and the need to prepare the groundwork of all the stakeholders involved in advance. This essay laid out the framework of Islamic bioethics and how it can be reflected onto the future discussions on neuroethics. The field of neuroethics and its increasing prominence permeates through numerous aspects of the human experience and social structures, as can be observed by the gradual increase of brain defense cases in legal courts. The matured deliberations by the collective efforts of Muslim religious scholars and scientists on various bioethical issues, such as cloning and genomics, as employed in this essay highlights that these topics may assist in predicting the trajectories of Islamic neuroethics and the challenges that might arise from within it.
